# Biochanin A attenuates doxorubicin-induced cardiotoxicity in rats with associated modulation of PI3K/Akt/mTOR and p38 MAPK signaling

**DOI:** 10.3389/fcvm.2026.1843282

**Published:** 2026-07-07

**Authors:** Mashael M. AlMutairi, Huda M. AlKreathy, Rasheed A. Shaik, Amani E. Alharbi, Rania Magadmi

**Affiliations:** 1 Department of Clinical Pharmacology, Faculty of Medicine, King Abdulaziz University, Jeddah, Saudi Arabia; 2 Department of Pharmacology and Toxicology, Faculty of Pharmacy, King Abdulaziz University, Jeddah, Saudi Arabia; 3 Department of Pharmacology and Toxicology, College of Pharmacy, Taibah University, Madinah, Saudi Arabia

**Keywords:** anti-apoptotic, anti-inflammatory, antioxidant, biochanin A, cardiac injury, cardiotoxicity, doxorubicin

## Abstract

**Background:**

Doxorubicin (DOX) is an effective anthracycline chemotherapeutic agent, but its clinical use is limited by dose-dependent cardiotoxicity associated with oxidative stress, inflammation, and apoptosis. Biochanin A (BCA), an O-methylated isoflavone, has demonstrated antioxidant and anti-inflammatory properties.

**Objectives:**

This study investigated whether BCA could mitigate DOX-induced cardiac injury in rats and explored the underlying molecular mechanisms.

**Methods:**

Male Wistar rats were randomly assigned to control, BCA-alone, DOX-alone, and DOX combined with BCA (25 or 50 mg/kg) groups. Cardiotoxicity was induced by DOX administration. Electrocardiographic (ECG) parameters were recorded, and serum cardiac biomarkers (CK-MB, LDH, and troponin) were measured. Cardiac tissue was evaluated for oxidative stress markers, antioxidant enzyme activities, inflammatory mediators, and apoptotic gene expression. Histopathological examination was performed. The involvement of PI3K/Akt/mTOR, p38 MAPK, and PTEN signaling pathways was assessed using molecular analyses.

**Results:**

DOX administration caused significant ECG abnormalities, elevated serum cardiac biomarkers, increased lipid peroxidation, reduced antioxidant enzyme activities, enhanced inflammatory cytokine levels, and upregulated pro-apoptotic markers in cardiac tissue. Histological examination revealed marked myocardial degeneration. BCA treatment attenuated these alterations, with greater effects observed at the higher dose in several endpoints. It was associated with improved antioxidant status, reduced inflammatory and apoptotic marker expression, decreased p38 MAPK and PTEN immunoreactivity, and was associated with altered PI3K/Akt/mTOR and p38 MAPK pathway-marker immunoreactivity compared with the DOX group.

**Conclusions:**

Biochanin A alleviated acute DOX-induced cardiotoxicity in rats and was associated with modulation of oxidative stress, inflammatory, apoptotic markers, as well as, PI3K/Akt/mTOR and p38 MAPK pathway markers. BCA may therefore represent a potential cardioprotective adjunct strategy in acute anthracycline-associated cardiac injury.

## 1 Introduction

Cancer remains a major public health challenge worldwide and accounts for approximately one in six deaths each year. According to global estimates issued by the International Agency for Research on Cancer and the American Cancer Society, nearly 10 million cancer-related deaths occurred in 2022. Lung, breast, colorectal, and prostate cancers are among the most frequently diagnosed malignancies. In Saudi Arabia, cancer represents a significant and growing health burden. The most recent report from the National Cancer Center of the Saudi Health Council documented approximately 28,100 newly diagnosed cancer cases and nearly 13,400 cancer-related deaths in 2022, based on IARC's global database (1). According to the American Cancer Society, breast, prostate, and colorectal cancers are among the most commonly diagnosed cancers in the United States in 2024 (2). These statistics underscore the continuing need for effective and safer anticancer therapies.

Among the available chemotherapeutic agents, doxorubicin (DOX), an anthracycline antibiotic introduced in the 1960s, remains a cornerstone chemotherapeutic agent for the treatment of a wide spectrum of malignancies, including breast cancer, bronchogenic carcinoma, lymphomas, neuroblastoma, gynecological cancers, Wilms’ tumor, thyroid cancer, testicular tumors, bladder cancer, and gastric cancer (3, 4). Over recent decades, DOX-containing regimens have substantially improved overall survival outcomes, with reported five-year survival rates reaching approximately 80% in certain cancers (5). Anthracyclines are approved by the U.S. Food and Drug Administration and are included in the World Health Organization's list of essential medicines due to their established therapeutic efficacy (6, 7).

Mechanistically, the antineoplastic activity of anthracyclines primarily arises from DNA intercalation and inhibition of topoisomerase II. By inserting between DNA base pairs, these agents interfere with replication and transcription processes (8). Topoisomerase II, particularly the α-isoform expressed in proliferating and malignant cells, is a critical molecular target mediating anthracycline-induced cytotoxicity (8–10). In addition, DOX promotes excessive generation of reactive oxygen species (ROS), leading to oxidative injury of lipids, proteins, and nucleic acids (11–13).

However, despite these therapeutic benefits, DOX is associated with significant off-target toxicity, particularly cardiotoxicity. Both acute and chronic cardiac complications have been documented, and the risk increases with cumulative dosing (14). As cancer survival rates continue to improve, long-term cardiovascular sequelae have emerged as a critical clinical concern, driving the expansion of cardio-oncology research and the search for protective strategies that can preserve cardiac function without com-promising anticancer efficacy (15).

In this context, increasing attention has been directed toward naturally derived compounds with potential cardioprotective properties. Biochanin A (BCA) is a naturally occurring O-methylated isoflavone found predominantly in leguminous plants such as soybeans, alfalfa, chickpeas, and peanuts (16, 17). Growing evidence indicates that BCA exhibits diverse pharmacological activities, including antioxidant, antiinflammatory, antineoplastic, antimicrobial, antidiabetic, and antiobesity effects (18). BCA has also been identified as an inhibitor of fatty acid amide hydrolase (FAAH), an enzyme involved in endocannabinoid signaling and pain modulation (19).

At the molecular level, BCA influences several signaling pathways implicated in inflammation and cellular proliferation. It has been shown to suppress inducible nitric oxide synthase (iNOS), inhibit phosphorylation of p38 mitogen-activated protein kinase (p38-MAPK) and activating transcription factor-2 (ATF-2), and prevent nuclear translocation of NF-κB, thereby reducing inflammatory signaling (20). Additional investigations have demonstrated inhibitory effects on β-site amyloid precursor protein cleaving enzyme 1 (BACE1), suggesting potential therapeutic relevance in neuro-degenerative disorders (21). Neuroprotective properties of BCA have been reported in models of cerebral ischemia/reperfusion injury through modulation of p38-dependent pathways (22) and in β-amyloid-induced toxicity via mitochondrial apoptotic mechanisms (23). Hepatoprotective effects have also been observed in lipopolysaccharide/D-galactosamine-induced liver injury through activation of Nrf2 signaling and suppression of the NLRP3 inflammasome (24). A detailed summary of BCA's pharmacological mechanisms across cancer, metabolic, cardiovascular, and neurological conditions has recently been published (25, 26).

Given these diverse biological activities, its potential cardiovascular effects warrant particular attention. Importantly, accumulating experimental evidence supports the cardioprotective potential of BCA. Previous studies have demonstrated that BCA reduces oxidative stress and enhances sirtuin-1 (SIRT1) expression, thereby improving cardiac dysfunction in type 2 diabetic rats (27). BCA has also been shown to attenuate endotoxin-induced inflammatory signaling in human umbilical vein endothelial cells (28) and to exert antihyperlipidemic effects in streptozotocin-induced diabetic models (29). These findings collectively suggest that BCA may confer cardiovascular benefits through antioxidant and anti-inflammatory mechanisms. However, to date, limited information is available regarding the effects of Biochanin A in experimental models of DOX-induced cardiotoxicity. Therefore, the present study was designed to investigate the potential cardioprotective effects of BCA against DOX-induced cardiac injury in rats. Specifically, we employed a well-established acute high-dose doxorubicin model to investigate early myocardial injury responses.

## 2 Article types

Original Research Article.

## 3 Materials and methods

### 3.1 Study ethical consideration

The animal procedures were ethically approved by the Research Ethics Committee of Faculty of Pharmacy, King Abdulaziz University (PH-1445-36, dated 1st, August 2024), in full adherence with international ethical standards. The experiment's layout complied with the ARRIVE 2.0 recommendations.

### 3.2 Experimental design

Thirty adults male Wistar albino rats (200 ± 20 g) were obtained from the Animal House Facility, Faculty of Pharmacy, King Abdulaziz University. Animals were housed under controlled environmental conditions (22 ± 3 °C; 60%–70% humidity) with a 12-h light/dark cycle. Rats were maintained in groups of five per cage and had unrestricted access to standard laboratory chow (20% protein, 4% fat, 5% fiber, plus essential nutrients; Manufactured by Grain Silos & Flour Mills Organization, Riyadh, Saudi Arabia) and tap water. A one-week acclimatization period was allowed before initiating the experimental protocol. All experimental procedures were conducted between 9:00 a.m. and 12:00 p.m.

Animals were randomly assigned into five groups (*n* = 6 per group) using a computer-generated randomization sequence (GraphPad QuickCalcs). Randomization was performed by an investigator not involved in outcome assessment. Group allocation was concealed using coded identifiers, and treatment solutions were prepared and labeled by an independent investigator. Animals were housed according to treatment group (no mixed-group cages), and treatment syringes were coded to ensure that investigators responsible for treatment administration, data collection, and analysis remained blinded until completion of all measurements. Each rat received treatments via two routes according to group allocation: intraperitoneal (i.p.) injection and orally by gavage (p.o.) administration. The control group received normal saline i.p. and vehicle p.o. The BCA group received BCA (50 mg/kg, p.o.) and normal saline (i.p.). The DOX group received doxorubicin (20 mg/kg, i.p.) and normal saline (p.o.). The DOX + BCA (25 mg/kg) group received DOX (20 mg/kg, i.p.) together with BCA (25 mg/kg, p.o.), while the DOX + BCA (50 mg/kg) group received DOX (20 mg/kg, i.p.) together with BCA (50 mg/kg, p.o.).

Biochanin A (BCA; APC-322, Aktin Chemicals, Inc., Chengdu, China) was dissolved in normal saline containing 1% dimethyl sulfoxide (DMSO) for intragastric administration. The selected BCA doses (25 and 50 mg/kg) were prepared according to Bai et al. (30). Doxorubicin (DOX; 20075/0109, Accord, Austria) was dissolved in normal saline and administered intraperitoneally at 20 mg/kg, as described by AlQahtani et al. (31). This dosing regimen represents a widely used acute cardiotoxicity model in rats, designed to evaluate early toxic cardiac injury rather than chronic anthracycline cardiomyopathy (32, 33).

BCA or its vehicle was administered once daily for seven consecutive days. On Day 7, a single dose of DOX (20 mg/kg, i.p.) was administered to the designated groups. In co-treated groups, BCA was administered one hour prior to DOX injection. All treatments were delivered at a fixed volume of 10 mL/kg. Control and DOX groups received equivalent volumes of vehicle (normal saline containing 1% DMSO) via the intragastric route, as reported previously (31).

Seventy-two hours after DOX administration (Day 10), rats were anesthetized using ketamine (50 mg/kg, i.p.) and xylazine (5 mg/kg, i.p.) for electrocardiographic assessment. Blood was collected from the retro-orbital plexus, allowed to clot for 15 min, and centrifuged at 3000 rpm for 10 min at 4 °C to obtain serum (34).

Subsequently, animals were euthanized by decapitation. Hearts were excised, rinsed in ice-cold saline, and gently dried. Portions were fixed in 10% neutral buffered formalin for histological and immunohistochemical analyses. Additional samples were preserved in RNA Protect Tissue Reagent (Qiagen, Cat. No. 76106), and remaining tissues were snap-frozen in liquid nitrogen and stored at −80 °C until further analysis. Animals were monitored daily for clinical signs of distress, including reduced mobility, abnormal posture, labored breathing, inability to access food or water, and body weight loss exceeding 20%. These criteria were predefined as humane endpoints. No animals met these criteria, no mortality occurred during the experimental period, and no animals were excluded from analysis.

Primary endpoints were predefined as indices of cardiac injury, including electrocardiographic (ECG) parameters (PR interval, QT, QTc, and QRS amplitude), serum cardiac biomarkers (CK-MB, LDH, and troponin), and histopathological injury assessment. These parameters were considered the principal indicators of DOX-induced myocardial injury and the main efficacy outcomes.

Secondary endpoints included cardiac oxidative stress markers, inflammatory marker immunoreactivity, apoptosis-related gene expression, and pathway-marker immunoreactivity (PTEN, PI3K, p-Akt, mTOR, and p-p38 MAPK). These measures were evaluated to explore potential mechanistic associations underlying the observed injury and cardioprotective effects.

### 3.3 Electrocardiographic (ECG) recording

On Day 10, anesthetized rats (ketamine 50 mg/kg, 63037-136-25, Hikma Pharmaceuticals PLC, London; xylazine 5 mg/kg, VR-3364, Bioveta, Czech Republic) underwent ECG recording using a PowerLab 8/35 system (34). Core body temperature was maintained at 37.5 °C using a thermostatically controlled heating pad. Surface electrodes were inserted subcutaneously into the right forelimb, right hind limb, and left hind limb. ECG parameters evaluated included QT interval, corrected QT (QTc), PR interval (expressed in milliseconds), and QRS amplitude (expressed in millivolts). ECG parameters were interpreted according to established rodent electrocardiographic guidelines (35). ECG acquisition and analysis were performed by an investigator blinded to treatment allocation.

### 3.4 Determination of serum cardiac biomarkers

Serum concentrations of creatine kinase-MB (CK-MB; SEA479Ra), lactate dehydrogenase (LDH; SEB370Ra), and cardiac troponin I (cTnI; SEA478Ra) were quantified using sandwich enzyme-linked immunosorbent assay (ELISA) kits (Cloud-Clone Corp., Houston, TX, USA). All procedures were conducted strictly according to the manufacturers’ protocols. All biochemical measurements were conducted using coded samples, and investigators were blinded to group identity during analysis.

### 3.5 Biomarkers evaluation of oxidative stress markers

Cardiac tissues were homogenized (10% w/v) in ice-cold phosphate-buffered saline (PBS, pH 7.4) using a Glas-Col tissue homogenizer (Terre Haute, USA). Homogenates were centrifuged at 10,000 × g for 20 min at 4 °C, and supernatants were collected for biochemical analysis. Superoxide dismutase (SOD; SD 2521), CAT (CA 2517), and MDA (MD 2529) levels were determined using commercial kits (Biodiagnostics, Egypt), following the manufacturers’ instructions. Total protein content was measured using a bicinchoninic acid assay kit (355526, MyBioSource, Inc., San Diego, CA, USA), based on the colorimetric method originally described by Smith et al. (36).

### 3.6 Histopathological analysis

Formalin-fixed cardiac tissues were rinsed with tap water, dehydrated through graded alcohol series, cleared in xylene, and embedded in paraffin. Paraffin blocks were sectioned into 5-µm-thick slices using a sliding microtome. Sections were deparaffinized, rehydrated, and stained with hematoxylin and eosin (H&E; 51275, E4009, TTR012, 365548, Sigma-Aldrich, St. Louis, MO, USA), Masson's trichrome, and Sirius Red stains. Histological staining procedures were performed according to standard protocols described previously (37). For standardized evaluation, three non-consecutive sections per animal were analyzed. From each section, five randomly selected non-overlapping fields were examined. Image acquisition parameters (light intensity, exposure time, and magnification) were kept constant across all groups. Slides were examined under a Nikon light microscope (Tokyo, Japan) by a blinded pathologist. Digital images were anonymized and coded prior to analysis.

Immunohistochemical staining was performed using the avidin–biotin–peroxidase complex (ABC) method as originally described by Hsu et al. (38). Primary antibodies included cardiac tissue expression of tumor necrosis factor-α (TNF-α; mouse monoclonal, ab220210, Abcam®, Cambridge, UK), inducible nitric oxide synthase (iNOS; rabbit polyclonal, ab283655, Abcam®, Cambridge, UK), nuclear factor-κB (NF-κB; rabbit monoclonal, #8242, Cell Signaling Technology®, Danvers, MA, USA), interleukin-1β (IL-1β; rabbit polyclonal, ab283818, Abcam®, Cambridge, UK), phosphorylated p38 mitogen-activated protein kinase (p-p38 MAPK; rabbit monoclonal, #4511, Cell Signaling Technology®, Danvers, MA, USA), phosphatase and tensin homolog (PTEN; rabbit monoclonal, ab267787, Abcam®, Cambridge, UK), phosphatidylinositol 3-kinase (PI3K; rabbit monoclonal, ab225720, Abcam®, Cambridge, UK), phosphorylated protein kinase B (p-Akt; mouse monoclonal, sc-271966, Santa Cruz Biotechnology®, Dallas, TX, USA), and phosphorylated mammalian target of rapamycin (p-mTOR; mouse monoclonal, sc-293133, Santa Cruz Biotechnology®, Dallas, TX, USA). Goat anti-rabbit IgG (ab150077, Abcam®, Cambridge, UK) and goat anti-mouse IgG (ab97240, Abcam®, Cambridge, UK) were used as secondary antibodies. Immunoreactivity was quantified by measuring optical density (OD) using ImageJ software (version 1.8.0, NIH, Bethesda, MD, USA), and the mean OD value per animal was used for statistical analysis (39).

### 3.7 Quantification of apoptosis-related gene expression

Messenger RNA levels of Bax, Bcl-2, and caspase-3 were quantified by real-time polymerase chain reaction (RT-PCR). Total RNA was extracted using Triazole reagent and purified with the NucleoSpin RNA Mini Kit. Complementary DNA (cDNA) was synthesized using the Omniscript RT Kit (205113, Qiagen, MD, USA). Thermal cycling comprised sequential denaturation (>90 °C), annealing (50–60 °C), and extension (72 °C) steps to achieve exponential amplification of target sequences. The primer sequences were as follows: Bcl-2 forward 5′-TGATAACCGGGAGATCGTGA-3′ and reverse 5′-AAAGCACATCCAATAAAAAGC-3′; Bax forward 5′-CCTGAGCTGACCTTGGAGCA-3′ and reverse 5′-GGTGGTTGCCCTTTTCTACT-3′; Caspase-3 forward 5′-CTCGGTCTGGTACAGATGTCGATG-3′ and reverse 5′-GGTTAACCCGGGTAAGAATGTGCA-3′; and β-actin forward 5′-TCCGTCGCCGGTCCACACCC-3′ and reverse 5′-TCACCAACTGGGACGATATG-3′. β-Actin was used as an internal housekeeping control to normalize gene expression levels. Primer design and experimental procedures followed Hareeri et al. (40).

Reverse transcription reactions contained RNase-free water, 10 ×  TaqMan RT Buffer (1 μL), 25 mM MgCl2 (2.2 μL), 2.5 mM dNTP mix (2 μL), random hexamers (0.5 μL), RNase inhibitor (0.2 μL; N8080119, Thermo Fisher, UK), and MultiScribe Reverse Transcriptase (0.25 μL). RT-PCR amplification was carried out on an Applied Biosystems StepOne real-time PCR system with the following cycling conditions: 25 °C for 10 min (incubation), 48 °C for 30 min (reverse transcription), and 95 °C for 5 min (enzyme inactivation). Melting curve analysis was performed to confirm primer specificity, and gene expression data were processed using the Applied Biosystems real-time PCR software. Amplification efficiencies for each primer pair was determined using standard curve analysis and was within the acceptable range (90%–110%). Relative mRNA expression levels were calculated using the 2^−*ΔΔ*Ct method, with β-actin serving as the endogenous reference gene, as described by Livak and Schmittgen (41). Fold changes were expressed relative to the control group. RNA samples were labeled using coded identifiers, and gene expression analysis was conducted prior to decoding group allocation to ensure investigator blinding.

### 3.8 Statistical analysis

Data are presented as mean ± standard deviation (SD) for six animals per group. Statistical comparisons were performed by one-way analysis of variance (ANOVA) followed by Tukey's *post hoc* multiple comparison test to assess differences among all experimental groups. GraphPad Prism 8.1 (GraphPad Software, La Jolla, CA, USA) was used for all analyses. A significance threshold of *P* < 0.05 was applied. Quantitative image analysis was conducted using ImageJ (version 1.8.0, NIH, Bethesda, MD, USA). Statistical analyses were conducted using coded datasets. Group allocation was revealed only after completion of statistical testing. All animals completed the study and were included in the final analysis, and no outlier exclusion was performed.

## 4 Results

### 4.1 Effects of BCA on DOX-induced electrocardiogram (ECG) abnormalities in treated rats

**Figure 1:**
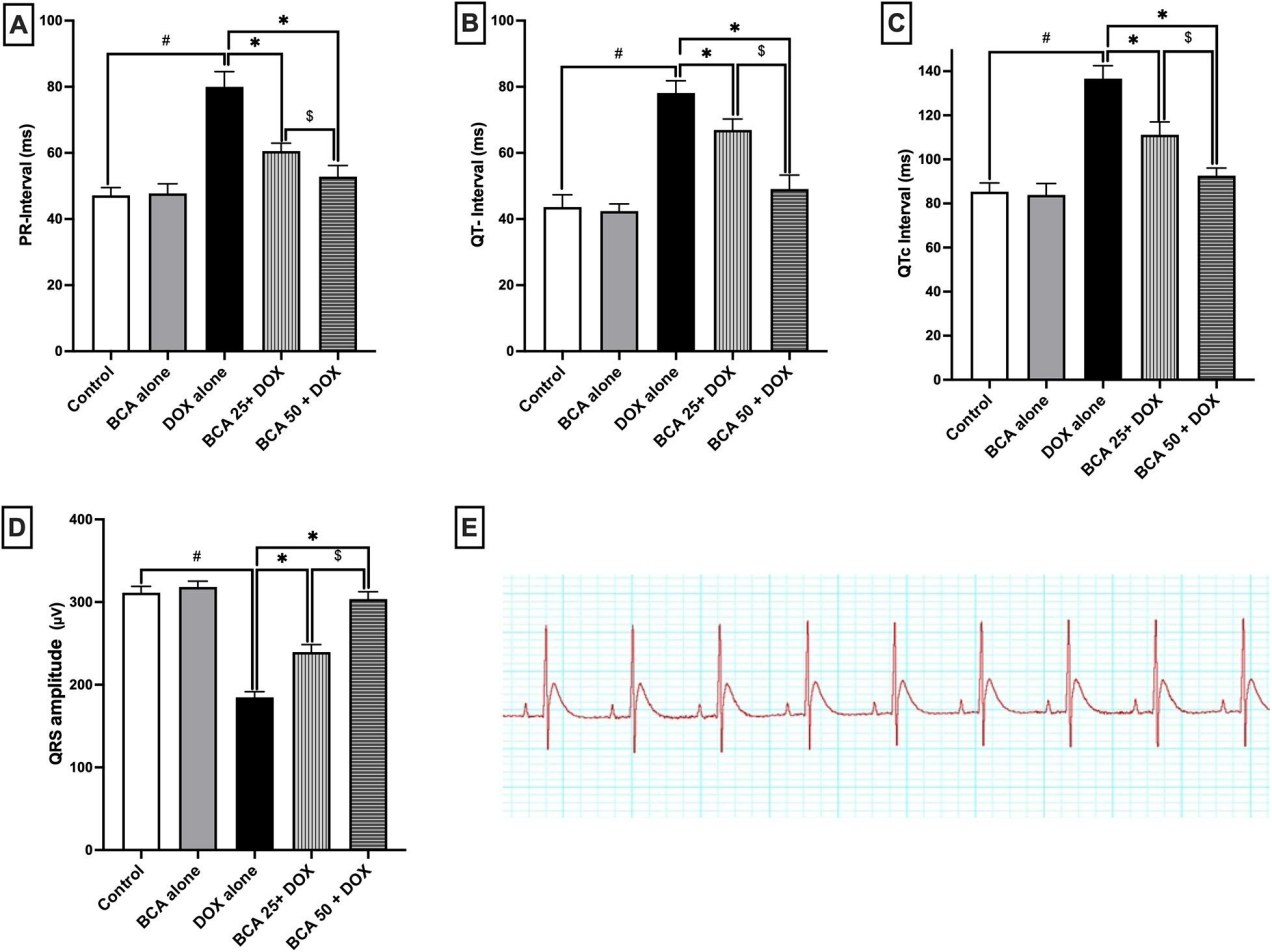
Effect of biochanin A (BCA) on doxorubicin (DOX)-induced electrocardiographic alterations in rats. **(A)** PR interval, **(B)** QT interval, **(C)** QTc interval, and **(D)** QRS amplitude measured in control, BCA-treated, DOX-treated (20 mg/kg, i.p.), DOX + BCA (25 mg/kg, p.o.), and DOX + BCA (50 mg/kg, p.o.) groups. **(E)** Representative ECG tracings from the control group. Data are expressed as mean ± SD (*n* = 6). Statistical analysis was performed using one-way ANOVA followed by Tukey's multiple comparison test. #*P* < 0.05 vs. Control group; *P < 0.05 vs. DOX group; $*P* < 0.05 vs. DOX + BCA (25 mg/kg) group. BCA: biochanin A, DOX: doxorubicin.

Electrocardiographic parameters, including PR interval duration, QT interval, corrected QT interval (QTc), and QRS amplitude, are presented in Figure 1.

Administration of DOX resulted in significant prolongation of the PR interval, QT interval, and QTc compared with the control group. In addition, DOX treatment markedly reduced QRS amplitude, indicating impaired electrical conduction and ventricular depolarization.

Co-treatment with BCA at both tested doses (25 and 50 mg/kg) significantly mitigated these DOX-induced ECG abnormalities. Notably, the higher dose of BCA (50 mg/kg) demonstrated a statistically greater corrective effect compared with the 25 mg/kg dose for selected ECG parameters, indicating dose-dependent improvement.

### 4.2 The effect of BCA administration on serum cardiac biomarkers in DOX-treated rats

**Figure 2:**
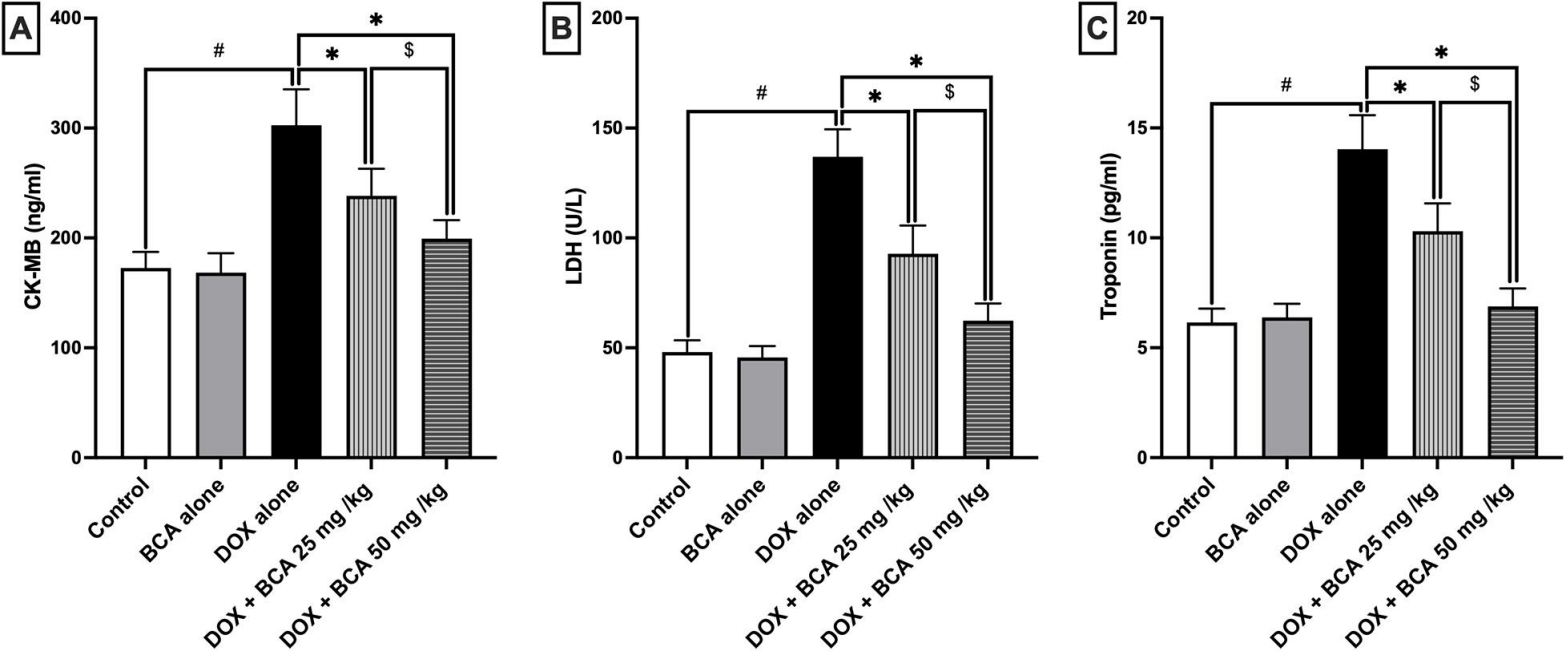
The effect of BCA on the serum levels of **(A)** CK-MB, **(B)** LDH, and **(C)** troponin of the rats treated with DOX. Data are expressed as mean ± SD (*n* = 6). Statistical analysis was performed using one-way ANOVA followed by Tukey's multiple comparison test. #*P* < 0.05 vs. Control group; *P < 0.05 vs. DOX group; $*P* < 0.05 vs. DOX + BCA (25 mg/kg) group. BCA: biochanin A, DOX: doxorubicin, CK-MB: creatine kinase-myocardial B fraction, and LDH: lactate dehydrogenase.

Exposure to DOX produced a marked increase in serum creatine kinase-MB (CK-MB) levels, with values rising by approximately 75% relative to the control group. Co-administration of BCA at doses of 25 and 50 mg/kg significantly mitigated this in-crease, lowering CK-MB concentrations by 21% and 34%, respectively, compared with the DOX-only group. The higher BCA dose exerted a more pronounced effect than the lower dose (Figure 2A).

Similarly, DOX treatment significantly elevated serum lactate dehydrogenase (LDH) levels, reaching approximately 1.85-fold of control values. Concurrent treatment with BCA (25 or 50 mg/kg) significantly counteracted this rise, reducing LDH levels by 32% and 55%, respectively, relative to the DOX group. Greater improvement was observed with the 50 mg/kg dose compared with the 25 mg/kg dose (Figure 2B).

Serum troponin concentrations were also markedly increased following DOX administration, showing a 129% elevation compared with controls. Combined treatment with BCA significantly attenuated this increase, producing reductions of 26% and 51% at doses of 25 and 50 mg/kg, respectively, relative to DOX alone. Notably, the higher BCA dose nearly restored troponin levels to those observed in the untreated control group and demonstrated superior efficacy compared with the lower dose (Figure 2C).

Importantly, administration of BCA alone did not result in any statistically significant alterations in CK-MB, LDH, or troponin levels compared with the control group, indicating that BCA did not adversely affect cardiac enzyme profiles under normal conditions during the short experiment period.

### 4.3 The effect of BCA administration on cardiac histopathological changes in DOX-treated rats

**Figure 3:**
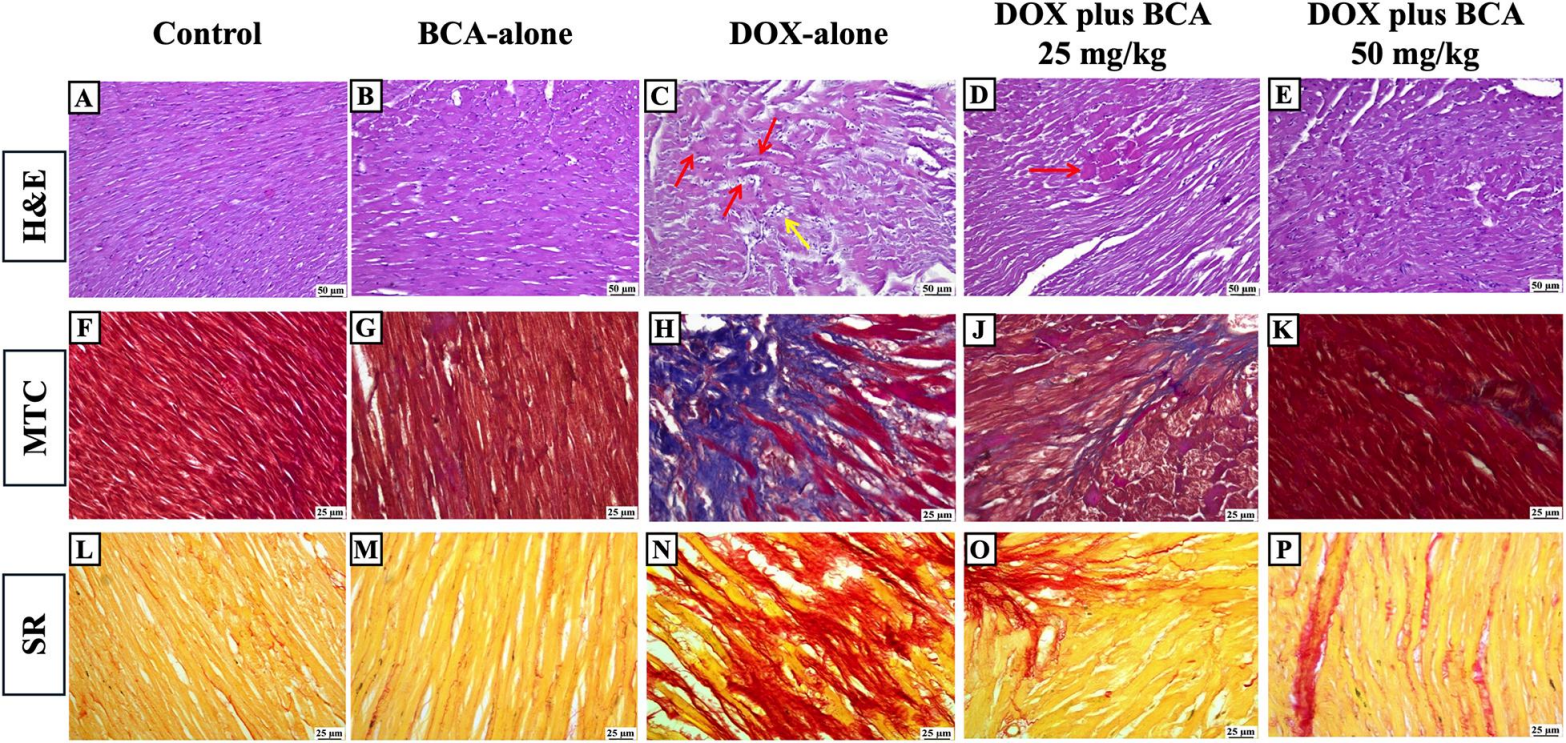
Representative photomicrographs of cardiac tissue sections stained with H&E **(A–E)**, masson's trichrome (MTC; **(F–K)**), and sirius Red (SR; **(L–P)**) in control, BCA-alone, DOX-alone, DOX + BCA (25 mg/kg), and DOX + BCA (50 mg/kg) groups. In H&E-stained sections, red arrows indicate interstitial edema and yellow arrows indicate cytoplasmic necrosis. In MTC-stained sections, collagen fibers appear blue. In SR-stained sections, collagen fibers appear red. Nuclear pyknosis is observed in DOX-treated sections.

Histopathological examination of the cardiac specimens stained with H and E, revealed a normal histological structure in the control group. The cardiac architecture was well-preserved, with organized myofibrils, intact myocytes, and no evidence of vacuolization, interstitial edema, or cellular damage. The nuclei appeared normal, with no signs of pyknosis or necrosis, indicating healthy cardiac tissue morphology (Figure 3A). In the group treated with BCA alone (50 mg/kg), the cardiac tissue structure was also normal, with no histopathological abnormalities observed. The cardiac architecture remained intact, and there were no indications of adverse effects from BCA administration (Figure 3B). In contrast, cardiac sections from the DOX-only-treated group exhibited pronounced histopathological changes consistent with cardiotoxicity. These changes included disorganization of myofibrillar morphology, vacuolization of myocytes, interstitial edema, cytoplasmic necrosis, and nuclear pyknosis (Figure 3C). However, rats treated with BCA, particularly at the higher dose (50 mg/kg), showed substantial restoration of normal cardiac tissue structure. The protective effects of BCA were evident in the preservation of cardiac morphology, indicating its potential to mitigate DOX-induced damage (Figures 3D,E).

Masson's trichrome (MTC) staining in the control and BCA-only groups showed minimal collagen deposition with preserved myocardial architecture, without evidence of abnormal extracellular matrix accumulation (Figures 3F,G). In contrast, cardiac sections from the DOX-only-treated group showed extensive blue staining, indicating increased collagen deposition suggestive of early extracellular matrix remodeling (Figure 3H). Treatment with BCA, especially at the higher dose, resulted in a marked reduction in the blue-stained areas compared to the DOX-only-treated group, indicating attenuation of DOX-induced collagen accumulation (Figures 3J,K).

Similarly, Sirius Red (SR) staining showed fine, organized collagen fibers in controls, stained red to indicate collagen presence (Figure 3L). The BCA-only group showed a similar pattern, with no excessive collagen deposition or abnormal extracellular matrix accumulation (Figure 3M). However, tissue sections from the DOX-only-treated group displayed abundant collagen deposition between cardiomyocytes (Figure 3N). Administration of BCA, particularly at the higher dose, significantly reduced the red-stained collagen fibers compared to the DOX-only-treated group (Figures 3O,P).

### 4.4 The effect of BCA administration on oxidative stress biomarkers in the hearts of DOX-treated rats

**Figure 4:**
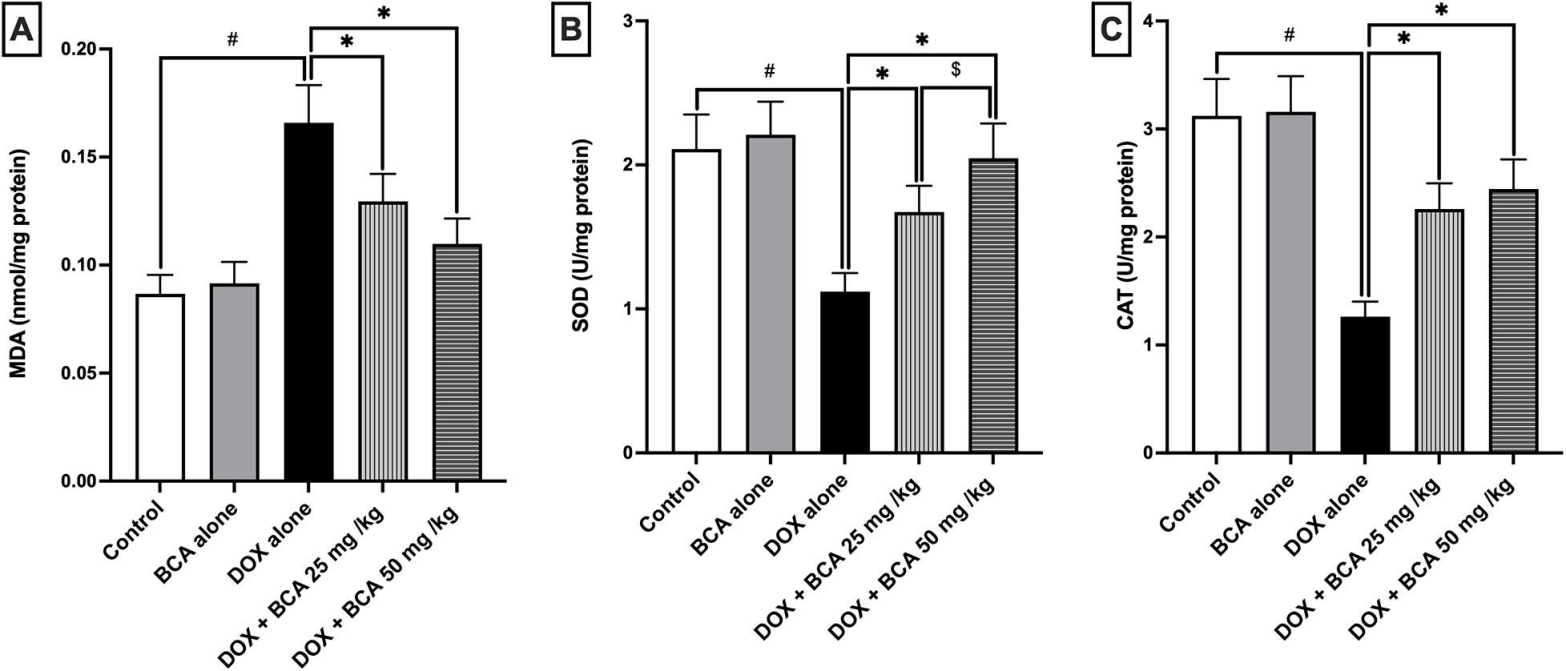
The effect of BCA on the cardiac tissue levels of **(A)** MDA, **(B)** SOD, and **(C)** CAT of the rats treated with DOX. Data are expressed as mean ± SD (*n* = 6). Statistical analysis was performed using one-way ANOVA followed by Tukey's multiple comparison test. #*P* < 0.05 vs. Control group; *P < 0.05 vs. DOX group; $*P* < 0.05 vs. DOX + BCA (25 mg/kg) group. BCA: biochanin A, DOX: doxorubicin, and MDA: malondialdehyde, SOD: superoxide dismutase, and CAT: catalase.

DOX exposure markedly enhanced oxidative stress in cardiac tissue, as reflected by a nearly twofold elevation in malondialdehyde (MDA) levels compared with the control group. Co-treatment with BCA at doses of 25 or 50 mg/kg significantly mitigated this increase, reducing cardiac lipid peroxidation by 24% and 35%, respectively, relative to the DOX-only group (Figure 4A). Despite the greater numerical reduction observed with the higher dose, the difference between the two BCA doses did not reach statistical significance.

In parallel, DOX administration led to a substantial decline in the activity of the antioxidant enzyme superoxide dismutase (SOD), with a 47% reduction compared with controls. Concomitant treatment with BCA significantly restored SOD activity. Relative to the DOX group, SOD levels increased by 49% and 88% following administration of 25 and 50 mg/kg BCA, respectively. The higher dose demonstrated a more pronounced restorative effect and resulted in SOD activity values approaching those of the control group (Figure 4B).

A similar pattern was observed for catalase (CAT) activity. DOX treatment significantly suppressed CAT activity by 59% compared with the control group. However, combined administration of BCA significantly reversed this suppression. CAT activity increased by 78% and 89% in the groups receiving 25 and 50 mg/kg BCA, respectively, compared with the DOX-only group (Figure 4C).

Importantly, rats treated with BCA alone exhibited no statistically significant alterations in MDA, SOD, or CAT levels compared with controls, indicating no significant alterations in cardiac oxidative stress markers during the short experimental timeframe.

### 4.5 The effect of BCA administration on the tissue expression of inflammatory biomarkers induced by DOX treatment in the hearts of treated rats

**Figure 5:**
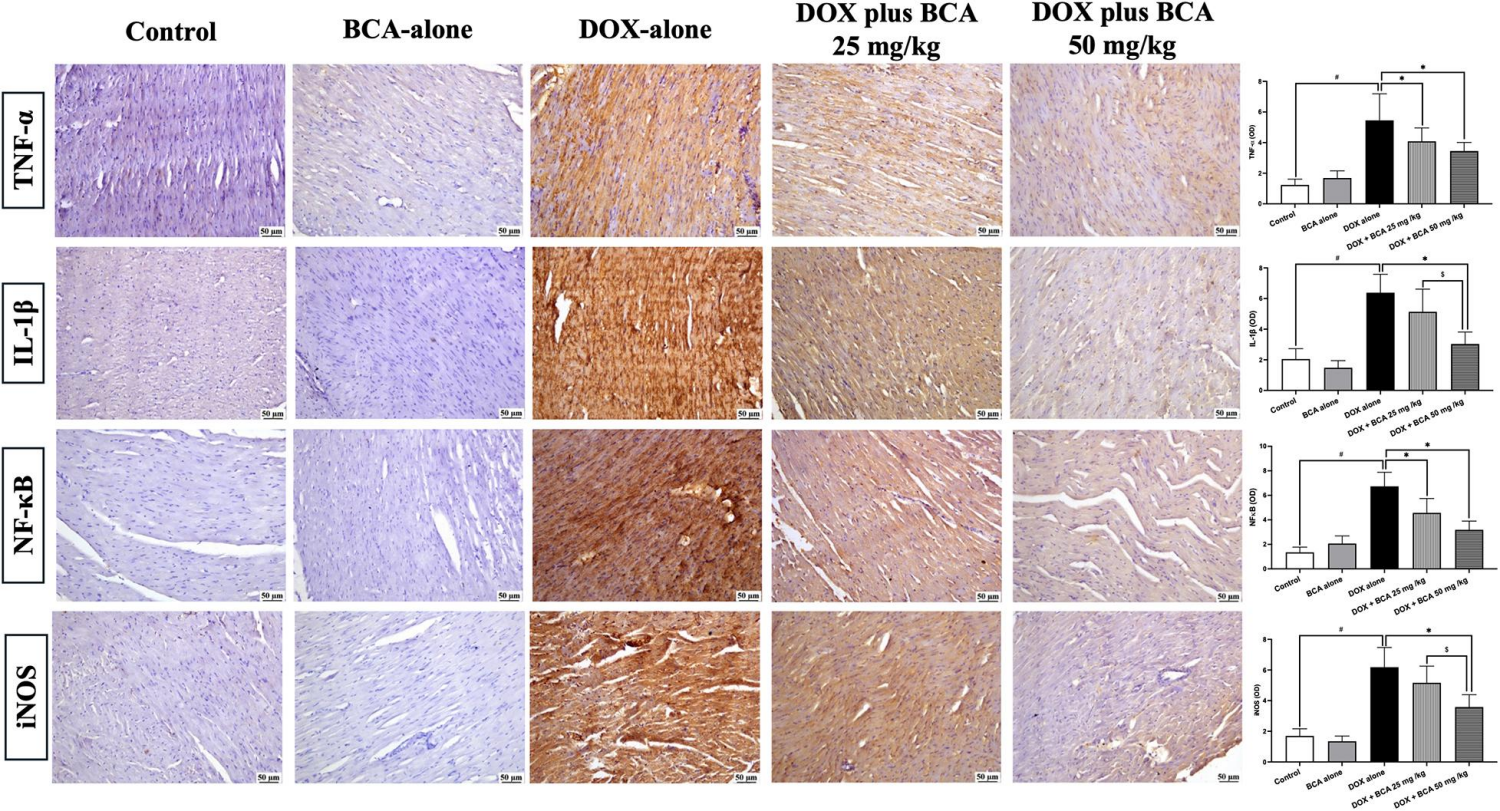
Effect of BCA on DOX-induced inflammatory marker expression in cardiac tissue. Representative immunohistochemical staining images of TNF-α, IL-1β, NF-κB, and iNOS expression in cardiac tissue sections from Control, BCA-alone, DOX-alone, DOX + BCA (25 mg/kg), and DOX + BCA (50 mg/kg) groups. Brown cytoplasmic staining indicates positive immunoreactivity. Scale ba*r* = 50 μm. Bar graphs represent quantitative analysis of immunoreactivity. Data are expressed as mean ± SD (*n* = 6). Statistical analysis was performed using one-way ANOVA followed by Tukey's multiple comparison test. #*P* < 0.05 vs. Control group; *P < 0.05 vs. DOX group; $*P* < 0.05 vs. DOX + BCA (25 mg/kg) group. BCA: biochanin A, DOX: doxorubicin, TNF-*α*: tumor necrosis factor-alpha, IL-1β: interleukin-1 beta, NF-κB: nuclear factor-kappa B, and iNOS: inducible nitric oxide synthase, and OD: optical density.

Immunohistochemical analysis revealed a pronounced inflammatory response following DOX administration. Cardiac tumor necrosis factor-α (TNF-α) immunoreactivity increased more than threefold compared with the control group. Co-treatment with BCA at 50 mg/kg markedly suppressed this elevation, significantly reducing TNF-α expression relative to the DOX-only group (Figure 5).

A comparable pattern was observed for interleukin-1β (IL-1β). DOX exposure ap-proximately doubled IL-1β immunoreactivity compared with controls. Administration of the higher BCA dose (50 mg/kg) significantly mitigated this response, lowering IL-1β expression by 53% compared with the DOX-treated group. Moreover, the reduction achieved with 50 mg/kg was significantly greater than that observed with the lower dose (25 mg/kg) and restored IL-1β levels to values not significantly different from those of the control group (Figure 5).

Similarly, DOX markedly upregulated NF-κB expression, with levels rising nearly 3.8-fold over control values. Both BCA doses significantly counteracted this increase. NF-κB expression declined by 31% and 52% in the groups receiving 25 and 50 mg/kg BCA, respectively, compared with the DOX-only group (Figure 5).

In addition, DOX treatment significantly enhanced the immunoreactivity of iNOS, showing a 264% increase relative to controls. Co-administration of BCA at 50 mg/kg significantly attenuated this elevation, reducing iNOS expression by 42% compared with the DOX group. The higher dose produced a significantly greater reduction than the 25 mg/kg dose (Figure 5).

Although the lower BCA dose (25 mg/kg) consistently exhibited a downward trend in TNF-α, IL-1β, iNOS, and NF-κB expression, these decreases did not reach statistical significance relative to the DOX group, with the exception of NF-κB, which was significantly reduced.

Importantly, rats treated with BCA alone showed no significant alterations in any of the assessed cardiac inflammatory markers compared with the control group, indicating no detectable adverse cardiac effects on the measured parameters during the short experimental period.

### 4.6 The effect of BCA administration on the apoptotic markers induced by DOX treatment in the hearts of the treated rats

**Figure 6:**
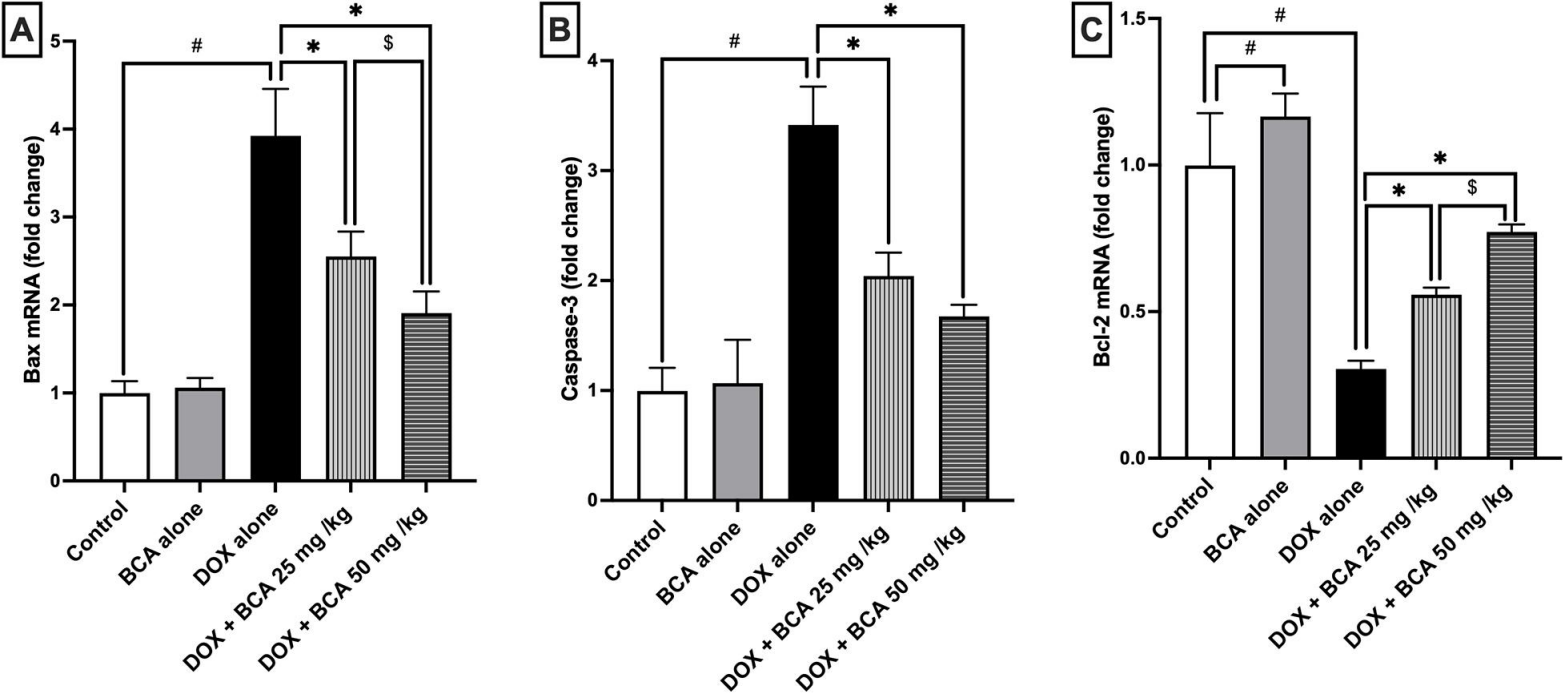
The effect of BCA on **(A)** Bax, **(B)** caspase-3, and **(C)** Bcl-2 genes expression in the cardiac tissue of the rats treated with DOX. Data are expressed as mean ± SD (*n* = 6). Statistical analysis was performed using one-way ANOVA followed by Tukey's multiple comparison test. #*P* < 0.05 vs. Control group; *P < 0.05 vs. DOX group; $*P* < 0.05 vs. DOX + BCA (25 mg/kg) group. BCA: biochanin A, DOX: doxorubicin, Bax: Bcl-2-associated X protein Bcl-2: B-cell lymphoma 2, and mRNA: ribonucleic acid messenger.

DOX exposure markedly promoted pro-apoptotic signaling in cardiac tissue, as evidenced by a nearly threefold elevation in Bax mRNA expression compared with the control group. Co-administration of BCA at both tested doses (25 and 50 mg/kg) significantly mitigated this upregulation in a dose-dependent manner. The higher dose produced a significantly greater suppression than the lower dose. Specifically, Bax mRNA levels were reduced by 33% and 51% in the 25 and 50 mg/kg BCA-treated groups, respectively, relative to the DOX-only group (Figure 6A).

A similar trend was observed for caspase-3 expression. DOX administration resulted in a 240% increase in caspase-3 mRNA compared with controls. Concurrent treatment with BCA significantly counteracted this elevation at both doses. The reductions in caspase-3 expression reached 41% and 50% in the groups receiving 25 and 50 mg/kg BCA, respectively, when compared with the DOX group (Figure 6B).

In contrast, the antiapoptotic marker Bcl-2 was markedly suppressed by DOX treatment, showing a 70% decrease relative to control values. BCA co-treatment significantly restored Bcl-2 expression in a dose- dependent manner. The higher dose exerted a significantly stronger effect than the lower dose. Compared with DOX-only rats, Bcl-2 mRNA levels increased by 87% and 157% in animals treated with 25 and 50 mg/kg BCA, respectively (Figure 6C).

Importantly, administration of BCA alone did not produce any statistically significant alterations in the expression of Bax, caspase-3, or Bcl-2 compared with the control group, indicating that BCA does not adversely influence basal apoptotic signaling in cardiac tissue during the short experiment period.

The Effect of BCA Administration on the p38-MAPK and PTEN/PI3K/Akt/mTOR Axis Alterations Induced by DOX Treatment in the Hearts of the Treated Rats.

**Figure 7:**
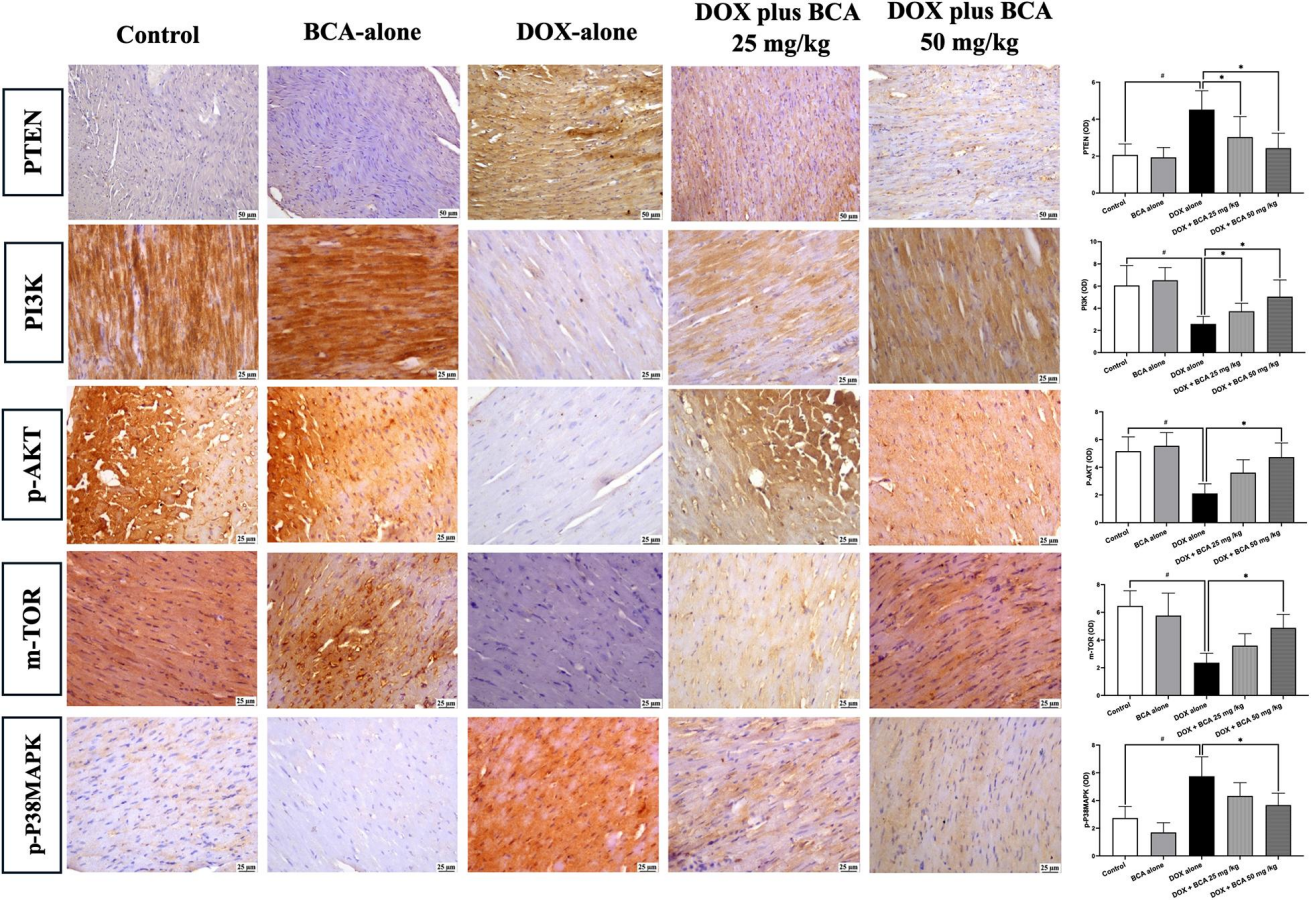
Effect of BCA on DOX-induced modulation of PTEN/PI3K/Akt/mTOR and p38 MAPK signaling pathways in cardiac tissue. Representative immunohistochemical staining of PTEN, PI3K, p-Akt, mTOR, and p-p38 MAPK in cardiac tissue sections from Control, BCA-alone, DOX-alone, DOX + BCA (25 mg/kg), and DOX + BCA (50 mg/kg) groups. Brown staining indicates positive immunoreactivity. Scale ba*r* = 25 μm. Bar graphs represent quantitative analysis of optical density (OD). Data are expressed as mean ± SD (*n* = 6). Statistical analysis was performed using one-way ANOVA followed by Tukey's multiple comparison test. #*P* < 0.05 vs. Control group; *P < 0.05 vs. DOX group. BCA: biochanin A, DOX: doxorubicin, PTEN: phosphatase and tensin homolog, PI3K: phosphoinositide 3-kinases, P-Akt: phosphorylated Akt kinase, mTOR: mammalian target of rapamycin, p-P38 MAPK: phosphorylated mammalian p38 mitogen-activated protein kinases, and OD: optical density.

Immunohistochemical analysis revealed alterations in the immunoreactivity of key components of the PTEN/PI3K/Akt/mTOR and p38 MAPK signaling pathways following DOX treatment. Immunohistochemical analysis demonstrated that PTEN expression was more than doubled in the DOX group relative to controls. Co-administration of BCA at 25 and 50 mg/kg significantly counteracted this elevation, lowering PTEN immunoreactivity by 33% and 47%, respectively, compared with the DOX-only group (Figure 7).

In contrast, DOX exposure substantially suppressed PI3K expression, showing a 57% reduction compared with the control group. Treatment with the higher BCA dose (50 mg/kg) significantly increased PI3K levels relative to DOX-treated rats, whereas the lower dose did not produce a statistically significant effect (Figure 7).

A similar pattern was observed for phosphorylated Akt (p-Akt). DOX administration resulted in a 59% decrease in p-Akt immunoreactivity compared with controls. Co-treatment with BCA at 50 mg/kg significantly increased p-Akt immunoreactivity compared with DOX-treated rats. Although the 25 mg/kg dose showed a tendency toward increased p-Akt expression, this change did not reach statistical significance (Figure 7).

Likewise, mTOR expression was significantly diminished following DOX treatment, with a 63% reduction compared with control values. The higher BCA dose (50 mg/kg) markedly increased mTOR immunoreactivity, increasing its expression by 104% relative to the DOX group. The lower dose produced a modest, but statistically non-significant, improvement (Figure 7).

Regarding stress signaling, DOX markedly enhanced p-p38 MAPK immunoreactivity, exceeding a twofold increase compared with the control group. Administration of BCA at 50 mg/kg significantly attenuated this activation, reducing p-p38 MAPK levels by 36% relative to DOX-treated animals. Although the 25 mg/kg dose exhibited a mild suppressive effect, the reduction was not statistically significant (Figure 7).

Importantly, rats receiving BCA alone did not exhibit significant alterations in any components of the assessed signaling pathways compared with controls, indicating no significant alterations in baseline cardiac signaling markers.

## 5 Discussion

Doxorubicin is an anthracycline chemotherapeutic widely used for treating various malignancies, including breast cancer, lymphomas, and sarcomas (42). Despite its remarkable efficacy, its clinical utility is significantly limited by its dose-dependent cardiotoxicity. Cardiotoxicity remains the most concerning adverse effect associated with anthracyclines, including DOX, and poses a challenge in long-term cancer management. This deleterious side effect often restricts the therapeutic potential of DOX, thereby necessitating strategies to mitigate its harmful impact without compromising its anticancer effectiveness (42). Acute manifestations include transient arrhythmias, and ECG abnormalities, whereas chronic toxicity is characterized by progressive left-ventricular dysfunction that can culminate in symptomatic heart failure (42). These clinical effects arise in part from excessive reactive oxygen species (ROS) generation, mitochondrial dysfunction, inflammation, and apoptosis within cardiac tissue (43, 44). Despite extensive investigation, the complete molecular mechanisms underlying these pathological effects remain incompletely understood.

Biochanin A (BCA) is an O-methylated isoflavone naturally occurring in leguminous plants such as soybeans, alfalfa, peanuts, and chickpeas (16, 17). Extensive evidence indicates that BCA exerts a broad spectrum of biological activities, including anti-inflammatory, antioxidant, neuroprotective, antimicrobial, anti-cancer, hepatoprotective, and cytoprotective effects (17, 45). In the cardiovascular context, BCA has demonstrated protective potential in several experimental models. It attenuated isoprenaline-induced myocardial fibrosis in mice, largely through modulation of oxidative stress (46), and improved cardiac abnormalities in diabetic cardiomyopathy by restoring redox balance and upregulating SIRT1 expression (27). However, no previous study has evaluated BCA in a DOX-induced cardiotoxicity model. Accordingly, the present study investigated the protective effects of BCA in a rat model of DOX-induced cardiac injury. In the present study, BCA was administered at 25 and 50 mg/kg. Using standard body surface area normalization (Km factor: rat = 6; human = 37), these doses correspond to approximate human equivalent doses (HEDs) of 4.1 and 8.1 mg/kg, respectively, which translate to approximately 246 mg and 486 mg per day for a 60-kg adult. It should be emphasized that HED estimation based on body surface area scaling does not establish achievable human plasma concentrations, myocardial exposure, metabolite profiles, or pharmacokinetic equivalence. The calculation provides only a theoretical dose translation and does not account for interspecies differences in absorption, metabolism, clearance, or tissue distribution.In the present study, DOX-induced cardiotoxicity was evidenced by significant ECG abnormalities, elevated serum cardiac biomarkers (CK-MB, LDH, and troponin), increased lipid peroxidation, reduced antioxidant enzyme activities, enhanced inflammatory marker immunoreactivity, upregulation of pro-apoptotic genes, and histopathological alterations including increased collagen deposition. These observations are consistent with previous studies describing oxidative stress, inflammation, apoptosis, and fibrotic remodeling as central features of DOX-induced cardiac injury, particularly in longer-term models (32–34, 47). The present study employed a single high intraperitoneal dose of doxorubicin (20 mg/kg), which represents a well-established acute cardiotoxicity model commonly used to investigate early oxidative, inflammatory, and apoptotic responses in preclinical studies (31–34, 44). While this model reliably induces measurable myocardial injury within a short experimental timeframe, it does not fully replicate the chronic cumulative cardiomyopathy observed clinically following repeated anthracycline exposure. Therefore, the conclusions of the current study primarily apply to acute DOX-induced cardiac injury. Future investigations utilizing chronic dosing regimens and long-term follow-up would further strengthen the translational relevance of these findings.

Administration of BCA attenuated ECG parameters, and reduced serum biomarkers of cardiac injury in a dose-dependent manner. These findings are in line with Mahajan & Goyal et al. (48), who investigated the mechanistic actions of BCA in a Streptozotocin (STZ) plus isopropyl (ISO)–induced diabetic myocardial infarction rat model. Further, the cardioprotective properties of BCA have been demonstrated in a rat myocardial ischemia/reperfusion injury model, which was established by transient ligation of the left anterior descending coronary artery followed by reperfusion (30). In this model, BCA administration significantly reduced myocardial infarct size and decreased serum levels of cardiac injury markers, including CK-MB, LDH, and aspartate aminotransferase. It should be noted that cardiac assessment in the present study relied primarily on ECG parameters and serum biomarkers of injury. Direct measurements of cardiac contractile performance, such as echocardiography or invasive hemodynamic indices (e.g., left ventricular ejection fraction or fractional shortening), were not performed. Therefore, the findings should be interpreted as reflecting attenuation of electrical activity and myocardial injury markers rather than definitive evidence of preserved global systolic function.

Histopathological examination in the present study further demonstrated preservation of myocardial architecture using H&E staining, along with reduced collagen accumulation as evidenced by Masson's Trichrome and Sirius Red staining. Similarly, Sharma et al. (46, 49) reported that BCA limited ISO-induced cardiac fibrosis in mice, decreased serum CK-MB levels, and reduced inflammatory cell infiltration and collagen deposition in cardiac tissues. Collectively, these findings are consistent with a potential cardioprotective effect of BCA in this acute experimental setting, as reflected by reductions in cardiac enzyme levels and improvement of histopathological alterations. In the current study, these effects were observed in a DOX-induced cardiotoxicity model and were supported primarily by ECG parameters and injury biomarkers.

DOX cardiotoxicity in this model was strongly associated with oxidative stress, as indicated by increased MDA levels and decreased SOD and CAT activities. These findings are consistent with the well-established role of mitochondrial dysfunction and ROS overproduction in DOX-induced myocardial injury (33, 44, 50, 51). Mitochondria are highly abundant in the myocardium, and DOX accumulation in mitochondria, redox cycling, and iron-mediated ROS generation contribute to lipid peroxidation and cellular damage (52). Further, DOX metabolites, including doxorubicinol, exacerbate the toxicity by increasing oxidative stress and mitochondrial dysfunction (53). Entering a vicious cycle of perturbations, such ROS-dependent damages induce more inflammation, which is fundamental in heart failure development (54, 55).

BCA significantly reduced lipid peroxidation and restored antioxidant enzyme activities, supporting its antioxidant potential. This effect may relate to its phenolic structure, which enables free radical scavenging (56). These findings are consistent with prior studies reporting restoration of antioxidant defenses by BCA in cardiac and metabolic injury models (17, 49).

Generally, inflammation, driven by pro-inflammatory cytokines and immune cell activation, contributes to the pathogenesis of cardiomyopathy, particularly in the context of drug-induced cardiac damage, leading to myocardial remodeling and fibrosis (47). Therefore, inflammation has been advocated as a central event in DOX-induced cardiotoxicity (51, 55). In the present study, DOX increased immuno-expression of TNF-α, IL-1β, NF-κB, and iNOS. Several reports demonstrated that DOX enhances the activity of iNOS, subsequently increasing the production of reactive nitrogen species (57). On the other hand, BCA-treated animals showed lower levels of the inflammatory marker's expression TNF-α, iNOS, NF-κB, and IL-1β. This aligns with the previously reported anti-inflammatory properties of BCA in cardiac tissues (49, 58). The attenuation of oxidative stress may contribute to reduced activation of inflammatory pathways (47, 51).

Apoptosis represents another key mechanism of DOX-induced injury (59, 60). DOX increased Bax and caspase-3 mRNA expression and reduced Bcl-2 expression. This is in line with previous reports highlighting a central role of apoptosis in DOX-induced cardiac injury (44). Our findings further support the concept that DOX-induced cardiotoxicity contributes to the pathogenesis of cardiomyopathy through oxidative stress, apoptosis, and extracellular matrix remodeling.

In this study, co-treatment with BCA demonstrated notable antiapoptotic properties that mitigated the toxic effects of DOX. These observations align with prior research that has reported the antiapoptotic effects of BCA in neurodegenerative and hepatotoxic models (61, 62). BCA has been reported to exhibit antiapoptotic effects in neuronal cells by modulating pathways such as PI3K/Akt and Toll-like receptor 4 (TLR-4)/NF-κB, reduce pro-apoptotic proteins (e.g., Bax, caspase-3) and enhance antiapoptotic ones (e.g., Bcl-2), thereby mitigating oxidative stress-induced apoptosis in models of neurodegeneration (63). This is in addition to the reported BCA antiapoptotic activities in experimental cardiac injury induced by cisplatin (64).

At the molecular level, several signaling pathways play pivotal roles in regulating the balance between survival and injury in cardiac tissue. The PTEN/PI3K/Akt/mTOR pathway is particularly important for maintaining cell growth, proliferation, metabolism, and survival. This pathway serves as a protective mechanism against stressors such as ischemia, reperfusion, and drug-induced injury (65). However, DOX has been shown to inhibit PI3K/Akt/mTOR signaling, contributing to cardiotoxic effects including oxidative stress, apoptosis, and cardiac dysfunction (66–69).

The mitogen-activated protein kinase (MAPK) pathway, which includes extracellular signal-regulated kinases (ERKs), p38 MAPK, and c-Jun N-terminal kinases (JNKs), is another critical regulator of cardiovascular pathology. The activation of p38 MAPK has been directly linked to apoptosis, and fibrosis in DOX-induced cardiotoxicity (70–72). Previous studies suggest that DOX-mediated activation of p38 MAPK and PTEN may contribute to suppression of PI3K/Akt/mTOR signaling and amplification of oxidative and inflammatory responses (66, 67, 69). In the present study, we observed alterations in the immunoreactivity of these pathway markers; however, causality cannot be established based on the current experimental design.

In the current study, BCA treatment was associated with alterations in pathway-marker immunoreactivity following DOX exposure; however, these findings should be interpreted as correlative rather than definitive evidence of pathway activation or inhibition. Specifically, BCA reduced p38 MAPK and PTEN immunoreactivity while increasing PI3K, p-Akt, and mTOR immunoreactivity compared with DOX-treated rats. These findings are consistent with previous reports demonstrating that BCA has been associated with alterations in PI3K/Akt–related signaling in experimental neuronal injury models, including rotenone-induced neurotoxicity and diabetic cerebral injury (73, 74). Furthermore, BCA's ability to inhibit p38 MAPK aligns with earlier studies reporting suppression of p38 MAPK signaling in cerebral ischemia/reperfusion injury models (22, 61).

**Figure 8:**
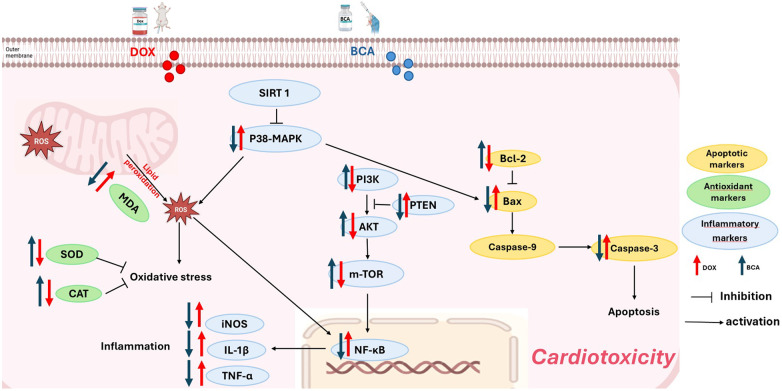
Hypothetical schematic model illustrating proposed associations underlying the protective effects of BCA in acute DOX-induced cardiotoxicity. BCA: biochanin A, DOX: doxorubicin; SOD: superoxide dismutase; CAT: catalase; MDA: malondialdehyde; TNF-α: tumor necrosis factor alpha; NF-κB: nuclear factor-kappa B; IL-1β: interleukin-1 beta; iNOS: inducible nitric oxide synthase; PTEN: phosphatase and tensin homolog protein; p-p38 MAPK: phosphorylated mammalian p38 mitogen-activated protein kinases; PI3K: phosphoinositide 3-kinase; Akt: phosphorylated Akt kinase; mTOR: mammalian target of rapamycin.

Collectively, the observed changes suggest that BCA administration may be associated with attenuation of several indices of DOX-induced cardiac injury in this acute model. The observed reduction in oxidative stress markers coincided with decreased inflammatory mediator expression and modulation of PTEN, p-p38 MAPK, PI3K, p-Akt, and mTOR immunoreactivity (Figure 8). However, it is important to interpret these signaling findings within methodological context. Pathway evaluation in the present study was based exclusively on immunohistochemical detection of selected pathway markers, including phosphorylated proteins. Although this approach enables spatial localization within preserved myocardial architecture and facilitates correlation with histopathological alterations, it remains inherently semi-quantitative. Importantly, total protein levels for Akt, mTOR, and p38 MAPK were not assessed in parallel, and therefore normalization of phosphorylated proteins to their corresponding total protein abundance (e.g., p-Akt/Akt, p-mTOR/mTOR, p-p38 MAPK/total p38 MAPK ratios) was not performed. Consequently, the observed differences in immunoreactivity cannot definitively distinguish altered phosphorylation state from changes in total protein abundance, altered tissue composition, variation in staining intensity, or secondary effects related to myocardial injury. Moreover, staining intensity does not directly reflect kinase activity or dynamic pathway flux. Accordingly, the reported alterations in PTEN, p-p38 MAPK, PI3K, p-Akt, and mTOR immunoreactivity should be interpreted as relative associations with BCA treatment rather than definitive evidence of functional pathway activation or inhibition. Future studies incorporating quantitative Western blot analysis with p-protein/total protein normalization, as well as functional modulation approaches, would provide more rigorous validation of mechanistic involvement.

The observed reductions in p38 MAPK and NF-κB immunoreactivity coincided with decreased collagen deposition and preservation of myocardial structural integrity in this acute setting. Activation of p38 MAPK and suppression of PI3K/Akt signaling have previously been linked to myocardial fibrosis, inflammatory remodeling, and adverse structural changes in experimental chronic cardiac injury models (47, 65, 71). Therefore, the present findings are consistent with previously reported pathway–fibrosis associations. However, because pathway activity was not functionally manipulated in the current study, these observations should be interpreted as correlative rather than definitive evidence of direct mechanistic regulation.

In this acute rat model of DOX-induced cardiotoxicity, BCA was associated with attenuation of ECG abnormalities, reductions in serum biomarkers of myocardial injury, and improvement of histological indices in this acute model. While these protective effects were accompanied by modulation of oxidative, inflammatory, apoptotic, and signaling markers, the findings should be considered preliminary within the constraints of the experimental design. Further investigation in chronic and clinically relevant anthracycline models is warranted. In addition, the relatively small sample size (*n* = 6 per group), although consistent with many exploratory preclinical cardiotoxicity studies (34), may limit statistical power and the generalizability of the findings, particularly for secondary molecular endpoints. Accordingly, the present results should be interpreted as preliminary and hypothesis-generating rather than definitive evidence of mechanistic pathway modulation or clinical cardioprotection.

From a translational perspective, several important considerations should be addressed before clinical application can be contemplated. First, the present study was conducted exclusively in male rats. Accordingly, the findings apply only to male animals under the described experimental conditions and should not be generalized to both sexes. Sex-dependent differences in anthracycline cardiotoxicity have been reported, and Biochanin A is an isoflavone with potential phytoestrogenic activity that may influence estrogen receptor–mediated signaling. Therefore, sex-specific differences in cardioprotective response are possible. Dedicated studies in female animals are required to determine whether hormonal milieu modifies the effects of BCA.

In addition, available evidence suggests that BCA exhibits moderate oral bioavailability and undergoes extensive hepatic metabolism, including demethylation to genistein and subsequent conjugation, which may influence systemic exposure and therapeutic consistency (17, 56). Therefore, detailed pharmacokinetic characterization and dose-optimization studies are required to determine systemic exposure, myocardial distribution, metabolite formation, and potential pharmacokinetic interactions with doxorubicin. The present study did not evaluate these parameters. Notably, this study assessed short-term cardiac parameters only and did not evaluate systemic toxicity markers such as liver or renal function indices, hematological parameters, body-weight trajectory, or long-term toxicity outcomes. Therefore, while no adverse cardiac effects were detected within the experimental timeframe, the present findings do not establish overall systemic safety of BCA. Furthermore, when proposing cardioprotective adjuncts in oncology, potential interference with the anticancer efficacy of doxorubicin must be carefully considered. The present study was conducted exclusively in healthy, non-tumor-bearing rats and did not evaluate tumor response, cancer cell cytotoxicity, or antitumor efficacy. Therefore, this study does not determine whether Biochanin A preserves, enhances, or compromises doxorubicin-mediated tumor cell killing. Although Biochanin A has demonstrated antiproliferative or chemosensitizing effects in certain experimental cancer models (45), these observations are presented solely as background information and should not be interpreted as evidence that the combination of Biochanin A with doxorubicin is oncologically safe. Direct evaluation in tumor-bearing models and cancer cell systems is required to establish whether cardioprotection occurs without attenuation of anticancer activity. This study provides initial experimental evidence regarding the effects of BCA in a male rat model of acute DOX-induced myocardial injury. Further studies are required to validate these findings in chronic models, tumor-bearing systems, and clinically relevant settings.

## Data Availability

The original contributions presented in the study are included in the article/Supplementary Material, further inquiries can be directed to the corresponding author.
